# Rosanna Peeling: improving diagnostic capacity of health systems

**DOI:** 10.2471/BLT.23.031023

**Published:** 2023-10-01

## Abstract

Rosanna Peeling talks to Gary Humphreys about the need for greater integration of diagnostics into health systems.

*Q: As a young post-doctoral researcher, you were invited by the Canadian government to establish a national reference laboratory for chlamydia. How did your career get off to such an impressive start?*

A: (laughing) I must have interviewed well! I also happened to be in the right place at the right time and had been working on the right pathogen. It’s also worth pointing out that I wasn’t so young, having taken time out after my master’s to raise two children. So perhaps there was a perception that I was mature enough for the role.

*Q: In what sense was it the right time?*

A: In 1991, the Canadian government was looking to establish a chlamydia screening programme. There had been some research revealing that *Chlamydia trachomatis* was the cause of sexually-transmitted as well as ocular infections; and since genital chlamydial infections are largely asymptomatic, screening programmes had to be set up for early detection and treatment to prevent sequelae such as ectopic pregnancy and tubal infertility. I was tasked with setting up the lab and using it to evaluate different diagnostic methods, considering their cost-effectiveness for both screening programmes and patient management. When I started out, all chlamydia screening was being done in clinics using time-consuming processes that included having to transport samples to labs for culture. Molecular methods, such as polymerase chain reaction (PCR), were just being commercialized. Although accurate, these methods were not very accessible, and it was about then that I started to take a keen interest in rapid point-of-care (POC) tests as a way of expanding access to diagnostics, and reinforcing what is of course a vital health system function – surveillance.

*Q: Can you explain how point-of-care tests work in simple terms?*

A: Three main diagnostic methods are used, the first being molecular tests which use nucleic acid amplification technology to detect a DNA or RNA target. The second is an antigen test, which is used to identify a specific protein from the pathogen such as the spike protein on the SARS-CoV-2 virus; and antibody tests, which detect the antibodies generated as part of the body’s immune response, providing an indirect indication of infection. All these methods are available in both laboratory-based and rapid POC test formats.

*Q: Which technique do you consider to be most effective?*

A: That’s an important question with far-reaching implications for public health policy. In my teaching, I like to talk about the “three As” that are crucial to diagnostics, namely: accuracy, affordability, and accessibility. From the accuracy point of view, the gold standard is the molecular test because of the sensitivity achieved through the amplification process. Molecular tests are also highly specific because they use the genomic sequence of the target pathogen. However, they are also expensive and demand specialized expertise to perform and interpret. Antigen tests are somewhat less sensitive, and some proteins may yield false-positive results because of cross-reactivity with other bacterial families. However – and this is an important consideration – antigen tests offer rapid results, often within 15 to 20 minutes, and are simple to perform. They are therefore highly accessible and affordable. The third category, antibody tests, reveal the body’s response to an infection. This may be useful information to indicate exposure, but not regarding current infection status, with the exception of some viral infections such as HIV.

*Q: There was much debate about the merits of PCR versus rapid antigen tests during the COVID-19 pandemic, with the latter often represented as ‘second best’. What is your view?*

A: It depends on how you define ‘best’. Best for what? The gold standard for diagnosing COVID-19 was certainly the PCR test, for the reasons I just gave, but the less-sensitive rapid antigen tests played a pivotal role in the pandemic response worldwide. While the PCR test made it possible to detect lower viral loads and thus catch infections earlier, this advantage was often negated by the protracted turnaround time of the PCR test result, especially in countries with limited PCR testing capacity. PCR tests also often showed positive for weeks and even months when people were no longer infectious. Though less sensitive, rapid antigen tests yielded a positive result that happened to be aligned closely with the transmission threshold for SARS-CoV-2. That meant that a positive result from an antigen test clearly indicated a need for self-isolation, which made them a very useful public health tool. So, there were significant trade-offs in the “three As” that were not always given sufficient consideration by policy-makers. I have seen similar biases among acknowledged experts, and sometimes came across them during my time at WHO.

*Q: Can you cite an example?*

A: I recall working on sexually transmitted diseases and organizing a meeting with a group of specialists to formulate target product profiles (TPPs) for diagnostics. We presented our TPPs to the prequalification team, who found that while our TPPs for chlamydia and gonorrhoea met the criteria for sensitivity and specificity, our TPP for syphilis, which proposed a sensitivity of 75%, did not. That level was grounded in an understanding that syphilis, being a mucosal infection, doesn't elicit a substantial antibody response, and our modelling had demonstrated that a test with 70% sensitivity could still significantly enhance infant survival rates. I appealed, and eventually prevailed. We ended up rolling out a simple finger-prick test which gave a result in 15 minutes and, in the case of a positive result, was followed by a shot of penicillin before the end of the second trimester. This was sufficient to prevent a baby from getting infected and dying. WHO later launched the Global Elimination of Congenital Syphilis Initiative using the same diagnostic approach. The tests have been shown to work in settings ranging from urban Brazil and China to remote East African villages. So, bottom line, the perfect should not be the enemy of the good. Fortunately, I would say that COVID-19 has fundamentally changed the discussion about diagnostics, putting us in a better place from a public health policy perspective.

*Q: Can you say more about that?*

A: During the first year of the pandemic, before vaccines and therapeutics were available, diagnostics emerged as the primary tool governments wielded to curb the virus’s spread. And they served a dual purpose: identifying cases for self-isolation or quarantine, and enabling the implementation of vital public health measures. Furthermore, they played a pivotal role in capturing the scale and trajectory of outbreaks, underpinning crucial research endeavours. All this informed an evolving recognition of their significance by governments, leading to a substantial allocation of resources to diagnostics – not the first time an emergency has helped advance the diagnostics agenda.

*Q: Do you have other examples?*

A: The 9/11 attacks in the United States of America (USA) prompted the government to invest in highly sensitive tests to detect potential bioterrorism agents. This led to the development of a POC molecular test using thermal cycling (a process used to amplify the target nucleic acid) that could be used in places like post offices, to identify threats like anthrax. This technology is now widely used for infectious diseases such as tuberculosis and HIV.

“Without diagnostics we are lost.”

*Q: Is the importance of diagnostics being reflected in ongoing discussions around pandemic preparedness?*

A: Absolutely, and I have been involved in several high-level consultations regarding pathogen preparedness, in which diagnostic capacity not only for patient care but also for disease control and surveillance is given full consideration. For example, last year, I participated in discussions with representatives of the G20 and USA and Canadian governments, where I presented the pressing need to establish a comprehensive diagnostic system in the aftermath of the pandemic, arguing that a connected diagnostic network was essential to healthcare systems, serving as their vigilant eyes and ears. The concept resonated strongly and attracted considerable support. Such networks can also play a key role in confronting the challenges posed by antimicrobial resistance (AMR).

*Q: Do you mean as a way of tracking the development of antimicrobial resistance?*

A: Yes, and better targeting of antimicrobial drugs. Presumptive administration of broad-spectrum antibiotics is no longer an acceptable approach if we want to preserve antibiotics for future generations. Treatment needs to be targeted, based on rapid, reliable diagnostics. Diagnostics can also detect outbreaks early, facilitating vaccine administration, which prevents infection and further development of resistant pathogens. Such a multifaceted synergy ­– employing vaccines, diagnostics and drugs – constitutes a comprehensive approach to tackling the AMR challenge head-on.

*Q: You are known for your work with the International Diagnostics Centre (IDC), which you set up in 2009 after leaving WHO. Can you talk about that initiative?*

A: My time at WHO brought me into contact with policy-makers, researchers, and the implementers of diagnostic programmes at country level. I got to see the profound impact of diagnostics – or lack thereof – on health-care systems in a wide variety of settings. It was an extraordinary learning experience, but it brought home to me the urgent need to collaborate with the private sector, something I could not do at WHO due to conflict-of-interest guidelines. So, I envisioned the IDC as a neutral platform, fostering collaboration between public and private sector stakeholders with a view to advancing our mission to assist countries. The IDC is now a worldwide network of centres committed not only to facilitating technological innovation, but to supporting the establishment of the kind of health systems needed to optimize the use of such tools. Because it’s all very well to get a reliable test, but what do you then do with the result? And that goes for health service delivery as well as surveillance. This brings me back to the idea of a connected diagnostic network being integrated into health-care systems.

*Q: What is next for you?*

A: I will continue in my roles as researcher, teacher and advocate, doing my best to get across the message that in a fast-changing world characterized by rapidly developing antimicrobial resistance and ecosystem change – with all the implications that has for population movement and emergent pathogens – we have never been in greater need of reliable, accurate, affordable and accessible diagnostics. Without them we are lost.

